# COVID-19 disease emergency operational instructions for Mental Health Departments issued by the Italian Society of Epidemiological Psychiatry

**DOI:** 10.1017/S2045796020000372

**Published:** 2020-03-31

**Authors:** Fabrizio Starace, Maria Ferrara

**Affiliations:** 1 Department of Mental Health and Drug Abuse, AUSL Modena, Modena, Italy; 2 Italian Society of Epidemiological Psychiatry (SIEP), Modena, Italy; 3 Department of Psychiatry, Yale University, School of Medicine, New Haven, CT, USA; 4 Program for Specialized Treatment Early in Psychosis, Connecticut Mental Health Center, New Haven, CT, USA

**Keywords:** Community mental health, emergency psychiatry, health service research, mental health, quality of care

## Abstract

During the current COVID-19 disease emergency, it is not only an ethical imperative but also a public health responsibility to keep the network of community psychiatry services operational, particularly for the most vulnerable subjects (those with mental illness, disability, and chronic conditions). At the same time, it is necessary to reduce the spread of the COVID-19 disease within the outpatient and inpatient services affiliated with Mental Health Departments. These instructions, first published online on 16 March 2020 in their original Italian version, provide a detailed description of actions, proposed by the Italian Society of Epidemiological Psychiatry, addressed to Italian Mental Health Departments during the current COVID-19 pandemic. The overall goal of the operational instructions is to guarantee, during the current health emergency, the provision of the best health care possible, taking into account both public health necessities and the safety of procedures. These instructions could represent a useful resource to mental health providers, and stakeholders to face the current pandemic for which most of Mental Health Departments worldwide are not prepared to. These instructions could provide guidance and offer practical tools which can enable professionals and decision makers to foresee challenges, like those already experienced in Italy, which in part can be avoided or minimised if timely planned. These strategies can be shared and adopted, with the appropriate adjustments, by Mental Health Departments in other countries.

## Background and rationale

During the current health emergency caused by COVID-19 disease in Italy (Livingston and Bucher, 2020) and worldwide, it is not only an ethical imperative but also a public health responsibility to keep the network of community psychiatry services operational, particularly for the most vulnerable subjects (those with mental illness, disability and chronic conditions) (Yang *et al*., 2020). In order to do so, it is necessary to share procedures that can be implemented in the whole Country (Italy). The goal is to avoid deepening the disparities of mental health resources within Regions or resistances to deliver appropriate mental health care.

The Italian Society of Epidemiological Psychiatry (SIEP) has compiled a list of practical instructions with two aims: (1) to reduce the spread of the COVID-19 disease within the outpatient and inpatient services affiliated with Mental Health Departments; (2) to write a document, which is fluid, and can be updated with the contributions of professionals working in Mental Health Departments (Società Italiana di Epidemiologia Psichiatrica, 2020). The overall goal is to guarantee, during the health emergency, the best health care possible, taking into account both public health necessities and the safety of procedures.

The Operational Instructions provided here report stricter rules regarding the use of personal protective equipment (specifically the use of fluid-resistant surgical masks) compared to the directions provided by the World Health Organization (World Health Organization, 2020). However, several lines of evidences support this decision, whenever supplies allow for its implementation. Subjects diagnosed with a mental illness have a life expectancy 15–20 years shorter compared to the general population (Nordentoft *et al*., 2013). The degree of risk of COVID-19 transmission within individuals with a severe mental illness is still unknown; however, it is reasonable to presume this risk is higher than that in the general population, because of persistent unhealthy behaviours and lifestyles. It is well known, for example, that this fragile population suffers from higher rates of respiratory diseases (Joukamaa *et al*., 2001) (because, in part, of the higher prevalence of cigarette smokers (Lasser *et al*., 2000)). Moreover, almost 15% of the patients currently treated by the community mental health care system had at least one admission to medium and long-term inpatient facilities during the previous year (Ministero della Salute, 2018), with the subsequent risk of acquiring nosocomial respiratory infections (Fukuta and Muder, 2013).

In light of the reasons mentioned herein and acknowledging the need to optimise the use of personal protective equipment (PPE), the following operational instructions are intended to protect mental health professionals, users and their families. We believe these recommendations, based on available evidences, are needed to ensure the fulfillment of the rights of this particularly vulnerable population during the current pandemic.

## Operational instructions for Mental Health Departments

### Outpatient activities

1. Phone check-ins will precede and/or replace scheduled outpatient visits. The assigned nurse will assess, through a phone call to the patient and/or family members, the patient's physical health status (presence of cough, fever *T* > 99.5 F, sore throat, shortness of breath) and mental health status (concerns about the current situation, changes in clinical symptomatology since last assessment). The health status of the family members will also be checked.
2. During the phone check-ins, the professional will provide information about (1) open hours, (2) changes in access to services and (3) public health recommendations about limiting social contacts. A final decision will be made whether to confirm the scheduled appointment (if deemed necessary) or reschedule it. Moreover, the mental health professional will stress the continued availability of acute services, in case of an emergency, during open hours (which will stay the same, resources permitting).
3. The scheduled appointment should be maintained in the following scenarios: (a) critical clinical situation, as assessed during previous visits or the phone check-in mentioned in point 1, and reported by the patient or caregivers (e.g. current exacerbation of symptoms, manifestation of new side effects, lack of adherence to the pharmacological treatment); (b) the necessity to administer pharmacological therapy at the centre (e.g. long-acting medications, direct observed therapy); (c) legal obligations (mandated to care).
4. Scheduled appointments can be postponed in the following scenarios: (a) clinically stable conditions; (b) patients with pre-existent vulnerable physical health conditions; (c) ascertained good adherence to treatment; (d) presence of supporting family; (e) preference expressed by the patient of phone/video call. If a patient is mandated to care (legal obligation), the designated professional will offer alternatives to in-person assessment to the judicial authority.
5. At the end of the phone call, the designated professional and the patient will agree on the frequency of follow-up phone/video calls which will be recorded in the electronic medical records and the professional's calendar. Services should implement an adequate telemedicine software both on telephones and laptops/computers accessible to all professionals.
6. The phone check-ins (point 1) can trigger a sense of abandonment to some of our patients. Please ensure that the patient understands that those phone calls are intended to reduce the risk of diffusion of the epidemic, and that the decision to postpone the scheduled appointment is shared by both the professional and the patient (if clinical conditions allow that).
7. Electronic medical records should report: name of the patient, professional who made the phone check-in, date of the next scheduled appointment, clinical notes. The head of the team should oversee that the above-mentioned procedures are carried out as instructed.
8. A list of daily accesses to the service for appointment that cannot be deferred and pharmacological therapy administration/pick up should be compiled, making sure to avoid crowding in the waiting area.

### Outpatient services. Preventive measures to increase the safety of patients and health care professionals

1. Front desk professionals must wear fluid-resistant surgical masks, use alcohol-based hand sanitiser and have constant access to cleaning products for wiping hard surfaces (e.g. desk).
2. Before entering the building, users will receive instructions on how to use hand sanitiser and will be redirected to the triage area by dedicated signs. Distance between users should be carefully maintained throughout all this whole process..
3. Users are asked to fill out a risk assessment form to check their current health conditions indicative of COVID-19 disease (cough, body temperature >99.5 F, shortness of breath) and if they have had contact with subjects at risk.
4. In the triage area, health care professionals at the front desk will check the information reported on the self-administered form and collect the following data: name and reason for visiting (e.g. scheduled appointment, acute-direct access, emergency).
5. Each center is asked to ensure a clear path to access the building, and will guide users from the triage zone to the waiting area.
6. In the waiting area, social distancing must be guaranteed (therefore, the number of people allowed should be carefully monitored, chairs can be moved/removed from the area).
7. Outside the triage area, signs will report the new procedure for access, and will remind users about the necessity to maintain interpersonal distancing while in line.
8. Accompanying people are discouraged to access the building. Exceptions are made for caregivers of subjects with severe physical disabilities. However, their presence in the waiting area should be taken into account in order to maintain the minimum interpersonal distance.
9. Before the in-person visit, users are encouraged to use hand sanitisers. During the visit, the minimal interpersonal distance is mandatory (3 ft), mental health professionals will wear fluid-resistant surgical masks, and the room will be ventilated whenever safe and appropriate. If the user shows signs of fever (*T* > 99.5 F) and/or shortness of breath, the mental health professional will provide a fluid-resistant surgical mask to the user while he/she will wear a long sleeve disposable apron and gloves.
10. Before performing a visit at home or at any other external facility, mental health professionals should take all the steps to minimise the risk of transmission through safe working procedures. Information on health conditions should be collected from the patient and family members. Mental health professionals will wear disposable apron, gloves and fluid-resistant surgical masks. Maintaining a minimal interpersonal distance (3ft) is mandatory. If the patient presents shortness of breath, a fluid-resistant surgical mask will be provided. Ventilate the room whenever safe and appropriate. Once the visit is completed, hand hygiene must be performed. If possible, home visit should be performed in open air. Minimal interpersonal distance should be maintained.
11. Group activities, both those for users and those for family members, are suspended. As an alternative, individual therapy sessions or family meetings can be provided, if necessary. Team leaders of those activities (e.g. group therapy) can follow up on a regular basis with regular participants to check clinical status and provide coping strategies.
12. Meetings are suspended. When necessary (e.g. multidisciplinary meeting involving different services for a vulnerable situation-discharge from hospital), meetings can be performed through telemedicine tools (such as video call).
13. In-person meetings preceding discharge from inpatient units should be performed following the same directions provided for home-visits.

### Day hospitals and day centres

These activities are suspended, as they favour large gatherings by definition. If it is not possible to suspend them, a drastic reduction of access to activities should be enforced in order to allow for preventive measures to reduce the risk of infection for patients, their families and mental health professionals. When suspended, the lead provider organises an alternative therapeutic programme (individual in person or phone/video sessions). It is suggested to increase the resources available to personnel (telephones, laptops) in order to guarantee regular follow ups.

### Short- and long-term residential care

These activities are carried out with the following regulations.

1. New admissions are suspended. Exceptions can be made for selected cases, as an alternative to a hospital inpatient admission or intensive outpatient psychiatric treatment (following a recent inpatient hospitalisation)
2. For new admissions, check the physical health status (specifically, the presence of cough, body temperature >99.5 F, sore throat, shortness of breath), and contacts at risk in the previous 14 days.
3. If the symptoms listed above are present, and the admission to the unit is deemed necessary, it is possible to admit the patient only if the facility can provide the following: rooms for isolation (single room with en-suite facilities), personal protective equipment, and a virus-screening test. If those requirements are not met, alternatives (home assistance included) where isolation and minimal but essential safety measures are provided should be considered.
4. Offer individual educational sessions to patients admitted to the unit, also providing printed materials, and encouraging hand hygiene. Check body temperature and respiratory rate daily.
5. Visitors are not allowed to the building. The director of the unit allows visitors only when those are deemed necessary. However, phone contacts should be favoured. Authorised visitors are required to wear a fluid-resistant surgical masks, will practice hand hygiene and will keep social distancing. Meetings should happen in open space, when possible.
6. Hospitalised patients are not allowed to go outside the unit. Exception should be authorised by the head of the unit.

### Inpatient units

Hospital inpatient admission to the psychiatric unit will continue, with the following adjustments.

1. Limit new admissions to clinical emergencies which cannot be deferred (e.g. compulsory admissions)
2. For new admissions, check the physical health status (specifically, the presence of cough, body temperature >99.5 F, sore throat, shortness of breath), and contacts at risk in the previous 14 days.
3. If the symptoms listed above are present or the person had a contact at risk in the previous 14 days, and the admission to the unit is necessary, it is possible to admit the patient only if the facility can provide the following: rooms for isolation (single room with en-suite facilities), personal protective equipment, and a virus-screening test. If those requirements are not met, evaluate the possibility to transfer the inpatient compulsory admission to a different facility (*TSO extraospedaliero* − compulsory admission outside the hospital (Ministero della Salute, 1978)) where isolation and minimal but essential safety measures can be provided.
4. Offer individual educational sessions to patients admitted to the unit, also providing printed materials, and encouraging hand hygiene. Check body temperature and respiratory rate daily.
5. Visitors are not allowed to the building. The director of the unit allows visitors only when those are deemed necessary. However, phone contacts should be favoured. Authorised visitors are required to wear fluid-resistant surgical masks, will practice hand hygiene, and will keep social distancing. Meetings should happen in open space, when possible.
6. Hospitalised patients are not allowed to go outside the unit. Exception should be authorised by the head of the unit.

## Conclusions

The operational instructions detailed above are aimed to guarantee, during the current COVID-19 disease emergency, the provision of the best health care possible, taking into account both public health necessities as the safety of procedures.

They will represent a useful resource to mental health providers, and stakeholders to face the current pandemic for which most Mental Health Departments worldwide are not prepared (Yang *et al*., 2020). These instructions will provide guidance and offer practical tools to enable professionals and decision makers to foresee challenges, already experienced in Italy, which in part can be avoided or minimized if timely planned. These strategies could be shared and adopted, with the appropriate adjustments, by other Mental Health Department.

## Appendix

Members of the board of the Italian Society of Epidemiological Psychiatry (SIEP) who contributed to the Operational Instructions include Francesco Amaddeo (Department of Neurosciences, Biomedicine and Movement Sciences, University of Verona), Walter Di Munzio (Salerno, Italy), Andrea Gaddini (Department of Public Health, ASL Roma2, Regione Lazio, Rome, Italy), Alessandro Guidi (Department of Mental Health and Drug Abuse, Azienda USL Toscana Nord Ovest, Italy), Lorenza Magliano (Department of Psychology, Campania University Luigi Vanvitelli, Caserta, Italy), Emiliano Monzani (Department of Mental Health and Drug Abuse, Bergamo Ovest, Italy), Antonella Piazza (Bologna, Italy), Elisabetta Rossi (Department of Mental Health AUSL Umbria 1, Italy), Giuseppe Tibaldi (Department of Mental Health and Drug Abuse, AUSL Modena, Modena, Italy).
